# Mending walls

**DOI:** 10.1186/1741-7007-10-41

**Published:** 2012-05-11

**Authors:** Gregory A Petsko

**Affiliations:** 1 Rosenstiel Basic Medical Sciences Research Center, Brandeis University, Waltham, MA 02454-9110, USA

## 

Before I built a wall I'd ask to know

What I was walling in or walling out

- *Robert Frost, 'Mending Wall'*

In rural New England, as in much of the rest of the world, people mark their territory, like some race of architecturally-adept spaniels, by building a wall around its borders. In some cases this is done for defensive purposes. In others, it is meant to keep in something that should not be allowed to roam freely (spaniels again, perhaps). But much of the time it is simply there to say, 'this is mine, not yours.'

Walls aren't inherently bad things, but in his classic poem on the subject, Robert Frost wonders if they might not have unintended consequences. Build a wall to keep something unpleasant out, and you are also walling yourself in, possibly with something else unpleasant. The point is that walls discourage human interaction, prevent the mixing of ideas, and create inbred - often xenophobic - cultures.

There is much talk today about the importance of interdisciplinary research, and much bemoaning of how difficult it is to carry it out, especially when the aim is to bridge the gap between basic discovery and translation to the clinic. One often hears the word 'silos' used to describe the separateness of the cultures. Silos is an evocative word, conjuring images of isolated white cylinders against the flat horizon of a Midwestern farm state; but I prefer to talk in terms of walls. Silos seem inherently separate. Walls can be knocked down.

Nowhere is the need for demolition more apparent than in the way biomedical research is conducted. There are 19 separate institutes at the National Institutes of Health (NIH), the largest financial supporter of biomedical research in the world. They are, for the most part, named for distinct diseases or organ systems (The National Cancer Institute; The National Eye Institute; The National Institute of Diabetes and Digestive and Kidney Disease; The National Heart, Lung and Blood Institute, and so on). They fund research in their own mission areas, and seldom venture outside the walls created by their specific name and charter, or their physical walls - they are generally housed in separate buildings on the sprawling NIH campus. Biomedical research funding reflects this separation: if you wish to apply for support for a cancer research project, you send your application to the National Cancer Institute, not to the National Institute on Aging, even though the risk for most cancers increases markedly with age. There are instances where multiple institutes and centers may come together to fund a project, but often they don't have the opportunity to do so, because the separateness of different areas of biomedical research is ingrained in the minds of the applicants, who target their proposals - and their investigations - accordingly.

And who would expect them to do otherwise? Go to any academic medical center in the US, or abroad, and you will find distinct departments of oncology for cancer research and treatment, rheumatology or immunology for arthritis and autoimmune diseases, neurology for Alzheimer's disease, psychiatry for schizophrenia, and so on - mirroring the disease - and organ-centeredness of the way we train medical specialists. Such divisions may make some sense in clinical care (though I wouldn't mind discussing that sometime), but increasingly, as we come to understand the cellular and molecular basis of disease, they make no sense in terms of research, basic or translational.

Parkinson's disease research is typically carried out in neurology departments. One would never expect to find Parkinson's researchers in departments of pediatrics or other places where inborn errors of metabolism are studied, and yet the biggest genetic risk factor for the development of Parkinson's disease is to be a carrier for the rare, autosomal recessive, lysosmal storage disorder called Gaucher disease. So tight is the connection that one expects virtually all Gaucher patients, who have two mutated copies of the relevant gene, to develop Parkinson's disease if they live long enough. The risk for the carriers, who have a single mutant copy, is almost ten times that of normal age-matched controls. Understanding the molecular basis for this surprising connection could lead to novel approaches to Parkinson's therapy (for example, could a drug to treat Gaucher disease reduce the risk of Parkinson's disease in Gaucher carriers down to normal levels?), but to do so will require smashing the wall between the very different medical disciplines that study and treat these seemingly different diseases.

Or consider the connections between obesity, diabetes, and cancer. Obesity frequently leads to type II diabetes, which makes sense in terms of the pathways that connect metabolism with insulin resistance. But diabetics also are at greatly increased risk for many forms of cancer - a connection that is much harder to explain (although some recent studies that revive the old idea of the Warburg effect and its importance for cancer cell metabolism may offer a partial explanation).

Lest you think that, at least, is starting to become clear, you might want to think again. Because if you examine the connection between diseases - what clinicians refer to as comorbidity - you run into paradoxes that cry out for more research. Obesity and diabetes are positively correlated with increased cancer risk? Not if you have schizophrenia.

Schizophrenics, in addition to myriad other problems, tend to have very unhealthy lifestyles. They are often chain smokers, and have high incidence of obesity and obesity-related diabetes. What they don't have is cancer. Cancer rates in schizophrenics are significantly lower than they should be given the positive comorbidity between cancer and obesity/diabetes in those who do not suffer from this mental illness. This inverse comorbidity even extends to lung cancer, which ought to be extremely high among schizophrenics given their dependence on cigarettes, and yet isn't.

I could list dozens more examples. (A favorite of mine is the inverse comorbidity between Parkinson's disease and nearly all cancers - with the striking exception of melanoma, which is so common among Parkinson's patients that neurologists have started to look for it. And if you're wondering whether the inverse is true, that melanoma survivors are at greatly elevated risk for Parkinson's, but that those who have had most other forms of cancer are at lower than normal Parkinson's risk, the answer is, that is indeed the case.) Every one of these connections offers a fascinating and, I think, fertile field for research. Yet such research is strikingly rare.

Balkanization of biomedical research funding by disease phenotype and organ system is one reason comprehensive studies of the connections between diseases are hard to find. Send a Parkinson's-melanoma grant to the National Institute of Neurologic Disorders and Stroke, and you are likely to find it sent back with a puzzled, 'Shouldn't this go to the National Cancer Institute?' Which, I suspect, would be equally puzzled by this grant that clearly is about neurologic diseases. But even if the funding agencies are more open-minded than that (and I suspect some of them might be), it's hard to imagine people trained in clinical research investigating such topics in the first place, because their training is very much disease-focused, and their research tends to be as well.

I believe we need a new way of thinking about disease. We have to get away from the classical emphasis on tissue, organ, and phenotypic presentation and think more about pathways and processes within the cell and organism. Seen this way, cancer is a disease of aberrant cell survival and Alzheimer's is a disease of aberrant cell death - should they not be inversely correlated? In fact, they are. Alzheimer's patients are at much higher risk relative to age-matched controls for developing Parkinson's disease - could the two disorders be intimately connected at the molecular level? There is increasing evidence that they are. It's difficult and expensive to do clinical trials on Parkinson's disease but much easier to do trials on Gaucher disease - once we realize they are connected, the rare disease may offer a route to the treatment of the more common one. More than half of all schizophrenic patients are not helped by the currently available treatment - perhaps we should look at some of the pathways that are involved in cancers for new targets and ideas.

Of course, none of this can happen without a change in the way biomedical research is organized and researchers are trained. There may be good reason from a clinical perspective to have separate departments of neurology and oncology, but given what I've told you, shouldn't they be in the same building, and shouldn't they have some joint seminars and grand rounds? Shouldn't the National Institutes of Health develop more mechanisms for cross-disease research funding? That would be a worthy objective for NIH's new National Center for Advancing Translational Sciences. And shouldn't NIH (and its foreign counterparts) also develop specific mechanisms to promote cross-disciplinary training? Not just for basic researchers, where the idea is already fashionable, but especially for physician-scientists. The biggest barrier to working across fields is the differences in jargon and mindset that each has. Overcoming that barrier is a job for education.

Translating discoveries from the concept to the clinic is hard, and it's getting harder as we tackle more chronic, non-infectious diseases (although the possibility that many of them may actually have an infectious etiology is something that bears remembering, and suggests yet another need for breaking down the walls between disciplines). We can't leave any stone unturned in our quest to improve human health, and some of the biggest unturned stones exist because we have built walls of specialization, paradigms, and tradition around the fields we work in.

Toward the end of his poem Robert Frost offers this observation:

*Something there is that doesn't love a wall*,

*That wants it down*.

When it comes to biomedical research, I think I know what that something is. It's called progress.

